# Bcl-2-mediated control of TRAIL-induced apoptotic response in the non-small lung cancer cell line NCI-H460 is effective at late caspase processing steps

**DOI:** 10.1371/journal.pone.0198203

**Published:** 2018-06-21

**Authors:** Lubna Danish, Dirke Imig, Frank Allgöwer, Peter Scheurich, Nadine Pollak

**Affiliations:** 1 Institute of Cell Biology and Immunology, University of Stuttgart, Stuttgart, Germany; 2 Institute of Systems Theory and Automatic Control, University of Stuttgart, Stuttgart, Germany; Institute of Biochemistry and Biotechnology, TAIWAN

## Abstract

Dysregulation of the mitochondrial signaling pathway of apoptosis induction represents a major hurdle in tumor therapy. The objective of the presented work was to investigate the role of the intrinsic (mitochondrial) apoptotic pathway in the non-small lung cancer cell line NCI-H460 upon induction of apoptosis using the highly bioactive TRAIL derivative Db-scTRAIL. NCI-H460 cells were TRAIL sensitive but an only about 3 fold overexpression of Bcl-2 was sufficient to induce a highly TRAIL resistant phenotype, confirming that the mitochondrial pathway is crucial for TRAIL-induced apoptosis induction. TRAIL resistance was paralleled by a strong inhibition of caspase-8, -9 and -3 activities and blocked their full processing. Notably, especially the final cleavage steps of the initiator caspase-8 and the executioner caspase-3 were effectively blocked by Bcl-2 overexpression. Caspase-9 knockdown failed to protect NCI-H460 cells from TRAIL-induced cell death, suggesting a minor role of this initiator caspase in this apoptotic pathway. Rather, knockdown of the XIAP antagonist Smac resulted in enhanced caspase-3 degradation after stimulation of cells with TRAIL. Of note, downregulation of XIAP had only limited effects on TRAIL sensitivity of wild-type NCI-H460 cells, but resensitized Bcl-2 overexpressing cells for TRAIL-induced apoptosis. In particular, XIAP knockdown in combination with TRAIL allowed the final cleavage step of caspase-3 to generate the catalytically active p17 fragment, whose production was otherwise blocked in Bcl-2 overexpressing cells. Together, our data strongly suggest that XIAP-mediated inhibition of final caspase-3 processing is the last and major hurdle in TRAIL-induced apoptosis in NCI-H460 cells, which can be overcome by Smac in a Bcl-2 level dependent manner. Quantitative investigation of the XIAP/Smac interplay using a mathematical model approach corroborates our experimental data strengthening the suggested roles of XIAP and Smac as critical determinants for TRAIL sensitivity.

## Introduction

Worldwide, lung cancer is the most common cause of cancer-related death in men and the third highest in women, being responsible for more than 1.5 million deaths in 2012 (World Cancer Report 2014, World Health Organization). Development of new treatment regimens for lung cancer like targeted therapy approaches is mandatory, because the success of conventional therapy is often limited due to acquired resistance [1]. Apoptosis is a tightly regulated form of controlled cellular self-destruction representing a major form of programmed cell death [2]. At the center of the cellular apoptotic program is a cascade of proteases, the caspases, the activation of which finally results in apoptosis. Caspases can be subdivided into a group of initiator caspases including caspase-2, -8, -9 and -10, and a group of executioner (effector) caspases including caspase-3, -6 and -7 [3]. Two main signaling pathways have been delineated to initiate the apoptotic program, called the extrinsic and the intrinsic pathway [4]. The extrinsic pathway is induced by activation of transmembrane receptors of the so called “death receptor” subgroup within the TNF receptor family which initiate apoptotic signals after binding their specific ligands. Activated death receptors recruit intracellular adapter molecules and form the death-inducing signaling complex (DISC) comprising procaspase-8/-10. These initiator caspases become subsequently cleaved and activated within the DISC. Once activated, they in turn cleave and activate downstream caspases, i.e. they initiate the caspase cascade.

The intrinsic apoptotic pathway is activated in response to signals resulting from severe cellular stress. Key event in this pathway is the permeabilization of the mitochondrial outer membrane (MOMP), whose integrity is mainly controlled by members of the Bcl-2 family. This large protein family consists of both pro- and antiapoptotic members which either induce or inhibit MOMP [5]. MOMP results in the release of soluble proapoptotic proteins into the cytosol, such as cytochrome c and second mitochondrial-derived activator of caspase (Smac/DIABLO). Cytochrome c initiates formation of the so-called apoptosome by promoting Apaf-1 oligomerization and triggering the activation of the initiator caspase-9, whereas Smac serves as a proapoptotic protein mainly by antagonizing the inhibitor of apoptosis (IAP) protein family member X-linked IAP (XIAP) [6].

In death receptor-mediated apoptosis two distinct cell types have been described, called type I and type II cells. In type I cells, caspase-8/-10 are directly and strongly activated within the DISC, allowing them to directly trigger a strong activation of the effector caspases caspase-3/-7 without a need for the involvement of the intrinsic pathway. On the other hand, in so-called type II cells, the mitochondrial pathway of apoptosis is required for amplification of the apoptotic signal [7]. In the center of this crosstalk of the two signaling pathways is the BH3-only protein Bid, becoming cleaved by activated caspase-8/-10 to truncated Bid (t-Bid). The latter then leads to activation of the proapoptotic Bcl-2 family members Bak and Bax within the outer mitochondrial membrane, resulting in MOMP allowing strong effector caspase activation and cell death [5].

The molecule TNF-related apoptosis-inducing ligand (TRAIL) is a member of the TNF ligand family and serves as a ligand for four cellular membrane receptors in the human system. Two of them, TRAILR1 and TRAILR2, are death receptors capable to activate the apoptotic program. The two other receptors, TRAILR3 and TRAILR4, are believed to mainly interfere with the proapoptotic effects of TRAIL by various mechanisms [8,9]. TRAIL is expressed on the cell surface as a type 2 transmembrane protein which can be released by proteolysis forming soluble TRAIL homotrimers. Interestingly, soluble TRAIL predominantly induces apoptosis in tumor cells, but not in normal tissues, and is well tolerated when given systemically [10]. However, clinical studies with soluble TRAIL so far showed only limited antitumoral effects [11]. Various attempts have been described to construct TRAIL-derived molecules possessing stronger antitumoral bioactivity. These include TRAIL molecules of higher valency, or fusion proteins with antibody derivatives for tumor targeting [11,12].

The human non-small cell lung carcinoma (NSCLC) line NCI-H460 has been characterized intensively in literature as a chemoresistant cell line [13,14]. In the present work we aimed at investigation of the underlying molecular mechanisms regulating the intrinsic apoptotic pathway of NCI-H460 in response to a highly potent TRAIL derivative, Db-scTRAIL [12]. This molecule possesses a higher valency (six versus three TRAIL monomers in a single molecule) and binds in addition with higher avidity due to targeting of epidermal growth factor (EGF) receptors with its antibody part. Db-scTRAIL is capable to also modify EGF signaling in EGF receptor (EGFR) positive and responsive cells, but the latter can be excluded in NCI-H460 cells, because these carry a KRAS mutation and have been shown to be unresponsive to EGF (M. Olayioye, unpublished data). Accordingly, in NCI-H460 cells Db-scTRAIL simply acts as a TRAIL derivative with enhanced affinity and receptor crosslinking activity, but without affecting EGFR signaling.

Here we show that Db-scTRAIL efficiently induces apoptosis in these cells in a mitochondria-dependent manner. Interestingly, apoptosis induction of these type II cells is strongly dependent on the mitochondrial release of Smac, rather than cytochrome c-mediated activation of caspase-9. TRAIL sensitivity/resistance in dependence of the expression level of Bcl-2 is crucially controlled by XIAP, the major antagonist of effector caspase-3. In line with these results, inhibition of apoptosis by Bcl-2 occurs at late steps of the proteolytic activation cascade of caspases. Applying mathematical models to analyze and predict responsiveness of cancer cells has emerged as a powerful tool [15,16]. We here adopt a mathematical description of the XIAP/Smac binding reaction to scrutinize our experimental results. In accordance with our data the model describes regions of TRAIL sensitivity and resistance, respectively, in NCI-H460, supporting the crucial role of the intrinsic pathway in apoptotic execution triggered by death receptor activation.

## Results

### Moderate Bcl-2 overexpression effectively protects NCI-H460 cells from TRAIL-induced apoptosis

**Fig 1:**
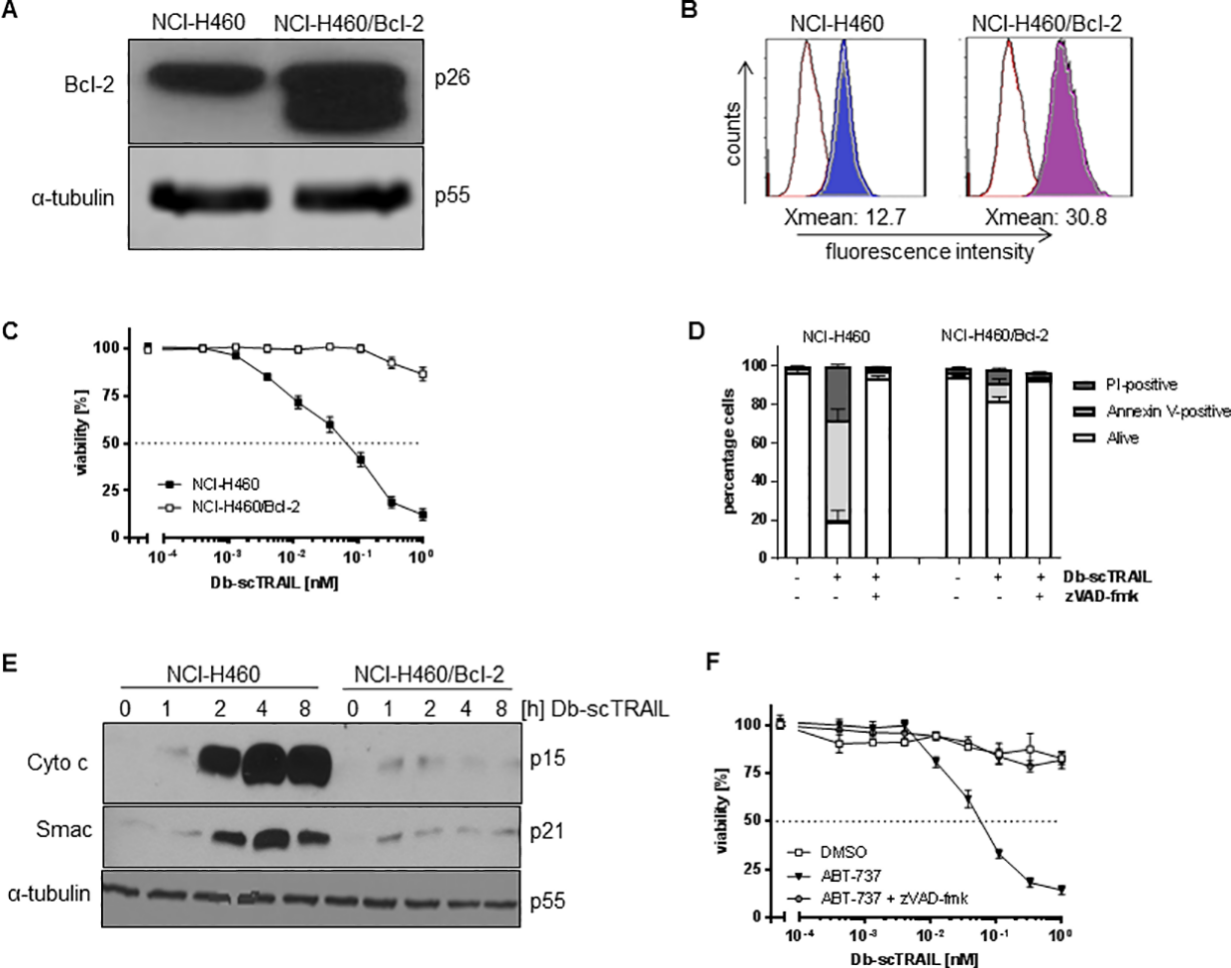
Moderate Bcl-2 overexpression effectively protects NCI-H460 cells from TRAIL-induced apoptosis. (A) Expression of FLAG-tagged Bcl-2 in NCI-H460 cells. Equal amounts of whole cell lysates from NCI-H460 wild type and NCI-H460/Bcl-2 were analyzed by immunoblotting. Note that the tagged Bcl-2 protein appears at a somewhat lower molecular weight range as compared to wild-type Bcl-2. Equal loading was confirmed by re-probing the membrane with α-tubulin specific antibody. (B) Detection of Bcl-2 by flow cytometry. Fixed cells were permeabilized and stained with anti-Bcl-2 or corresponding isotype control primary antibody and PE-labeled secondary antibody. Cells were analyzed by flow cytometry. (C) Cytotoxicity assays of NCI-H460 and NCI-H460/Bcl-2 cells using Db-scTRAIL. Cells were treated with increasing concentrations of Db-scTRAIL for 24 h, viable cells were stained with crystal violet and quantified at 550 nm. All values were normalized to values from unstimulated cells. Shown are mean values ± SD calculated from triplicates. Data shown is representative of three independent experiments. (D) Db-scTRAIL induces apoptosis in NCI-H460. Cells were co-treated with Db-scTRAIL and z-VAD-fmk (20 μM) or only the carrier DMSO for 24 h. Viable, apoptotic (Annexin V-FITC-positive) and necrotic (PI-positive) cells were determined after flow cytometric analysis. (E) Db-scTRAIL induces cytochrome c and Smac release into the cytosol in wild-type NCI-H460 cells only. Cells were left untreated or treated with Db-scTRAIL (0.5 nM) for the indicated time periods. Cytosolic extracts were prepared and subjected to western blotting with cytochrome c and Smac specific antibodies. Equal loading was confirmed by reprobing the membrane with α-tubulin antibody. (F) ABT-737 resensitizes NCI-H460/Bcl-2 cells. Cells were preincubated with 2.5 μM ABT-737 alone (filled triangle) or in combination with 50 μM z-VAD-fmk (filled circles) or DMSO as control (open squares) and treated with Db-scTRAIL. Viable cells were quantified by crystal violet staining after 24 h. Data represents mean values ± SD calculated from triplicates and shown is a representative of three independent experiments.

NCI-H460 cells have been described as type II cells in literature, representing cells where death receptor-induced apoptosis is dependent on the mitochondrial apoptotic pathway to amplify effector caspase activation [17]. The reason for this dependency is believed to be caused by a comparably weak activation of the initiator caspases-8/-10 within the DISC [7]. To analyze the relevance of the mitochondrial pathway for efficient apoptotic signaling in response to the dimerized targeted Db-scTRAIL, a subline of NCI-H460 stably overexpressing a FLAG-tagged Bcl-2 molecule (NCI-H460/Bcl-2) was established (Fig 1A). Note that the tagged Bcl-2 protein appears at a somewhat lower molecular weight range as compared to endogenous Bcl-2 suggesting that we overexpressed the smaller alternative spliced isoform β [18]. Overexpression of Bcl-2 was comparably low, we estimated from intracellular cytofluorometric analyses (Fig 1B) a factor in the range of about 3. Nevertheless this subline showed strong TRAIL resistance in comparison to wildtype cells (Fig 1C and 1D), confirming a cell type II for NCI-H460. This data already indicated that TRAIL receptor-mediated induction of apoptosis in NCI-H460 cells is a process quite sensitively regulated.

Control experiments confirmed that TRAIL-induced cell death is in fact apoptosis, because the broad range caspase inhibitor zVAD-fmk efficiently protected NCI-H460 cells (Fig 1D). Further, a strong release of the mitochondrially located molecules cytochrome c and Smac/DIABLO into the cytosol could be observed, beginning after about 2 h of TRAIL stimulation in wildtype NCI-H460 cells, but not in Bcl-2 overexpressing cells (Fig 1E). As expected, the latter cells, being highly unresponsive to TRAIL treatment *per se*, were effectively resensitized to a TRAIL stimulus in the presence of the cell permeable Bcl-2 inhibitor ABT-737 (Fig 1F). Again, cell death could be efficiently inhibited by the pan caspase inhibitor zVAD-fmk.

Overexpression of Bcl-2 showed no effects on the surface expression of TRAIL receptors, EGFR or on the expression levels of caspase-8, caspase-3 and XIAP (S1 Fig). Interestingly, when we analyzed the mitochondrial membrane potential (MMP) using TMRM as fluorescent probe, we observed a fraction of approximately 30% of all cells within the population showing reduced MMP (S1 Fig). Notably, Bcl-2 overexpression rescued this heterogeneity within the cell population. As differences in the MMP have been linked to apoptosis resistance [19,20], we monitored TMRM-stained cells by live-cell imaging and quantified the time from TRAIL treatment to death as a function of the cellular TMRM intensity for individual cells. However, we observed no correlation of the time to death values with the MMP (S1 Fig).

### Bcl-2 overexpression strongly inhibits caspase activity but affects caspase cleavage preferentially at late activation steps

**Fig 2:**
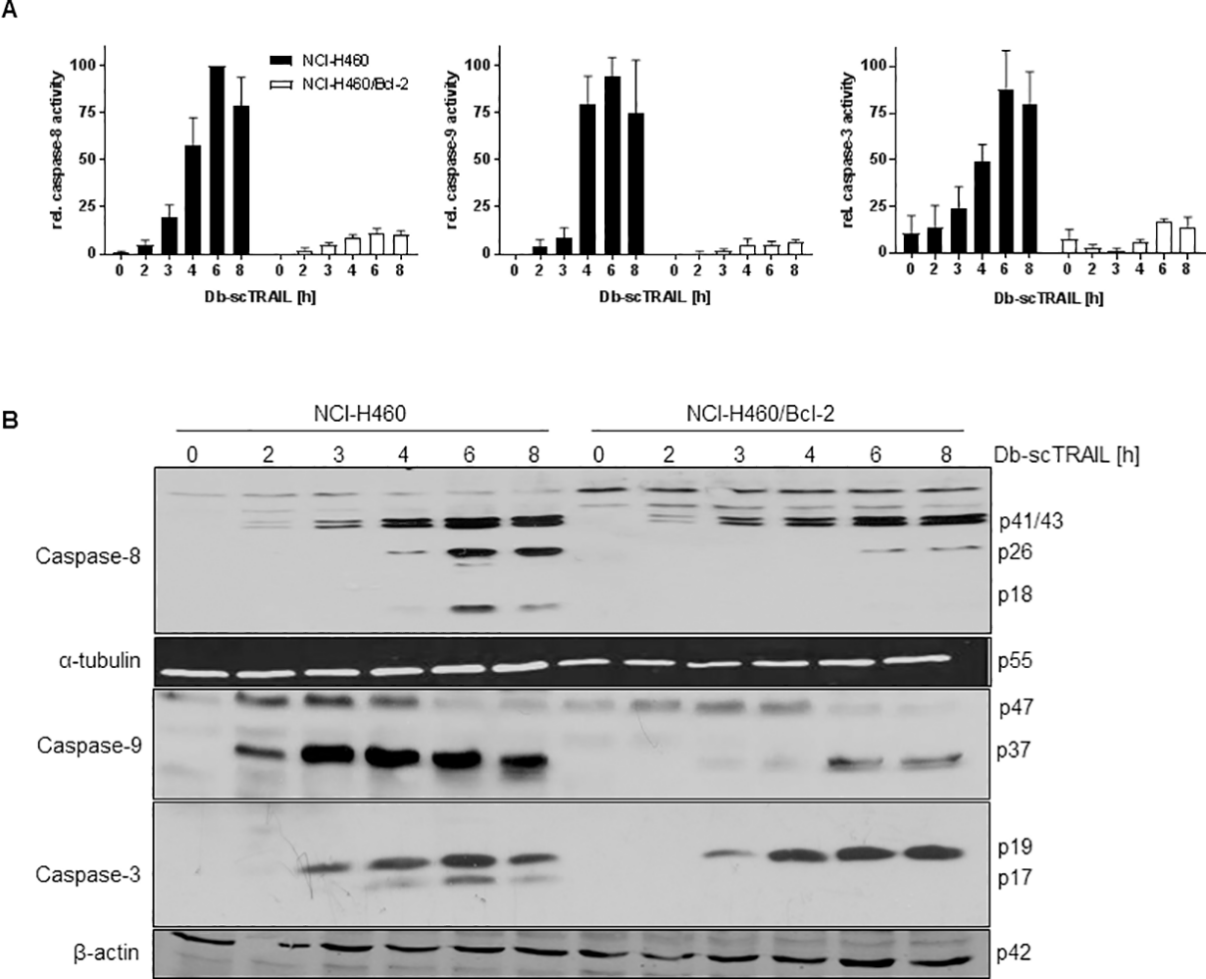
Bcl-2 overexpression strongly inhibits caspase activity but affects caspase cleavage preferentially at late activation steps. (A) Cells were stimulated with Db-scTRAIL (1 nM) for the indicated time periods. Whole protein lysates were incubated with fluorogenic caspase substrates Ac-IEPD-AMC (caspase-8), Ac-DMQD-AMC (caspase-3) and Ac-LEHD-AMC (caspase-9), respectively. Increasing fluorescence values were measured every 2 min for 2 h at λ = 460 nm. Data points shown, representing slopes, are mean values ± SD calculated from 3 independent experiments, normalized to the highest value of each experiment. (B) Cells were stimulated with Db-scTRAIL (1 nM) up to 8 h, one group was left untreated as a control. Equal amounts of whole cell lysates were subjected to immunoblot analysis using antibodies specific for cleaved caspase-8, cleaved caspase-3 and caspase-9 followed by HRP-conjugated secondary antibody.

Investigating the enzymatic activities of the caspases -8, -9 and -3 we found increasing values in response to TRAIL stimulation peaking after about 6 to 8 h of stimulation (Fig 2A). As expected, the enzymatic activities of all three caspases were strongly reduced in the cells overexpressing Bcl-2, which were effectively protected from TRAIL-mediated apoptosis (Fig 2A). Caspases become activated by sequential enzymatic cleavage steps, Western blotting experiments revealed distinct cleavage pattern as shown in Fig 2B. For caspase-8 the initial cleavage step to the p41/p43 products became visible after about two hours of TRAIL stimulation and their amounts increased up to about six hours of stimulation. Interestingly, this initial cleavage step, believed to occur within the death receptor signaling complex DISC [21], was comparable for NCI-H460 wildtype cells and the Bcl-2 overexpressing cells, strongly suggesting that p41/p43 production was not dependent on the mitochondrial signaling pathway, e.g. in terms of a caspase-9/caspase-3-mediated positive feedback on caspase-8 cleavage [22]. Clearly, however, the final caspase-8 cleavage, resulting in formation of the cytosolically located p18 product [23], could be detected in wildtype cells only, but not in NCI-H460/Bcl-2 cells (Fig 2B). In a similar way, the cleavage kinetics and pattern of caspase-3 to the p19 product was comparable for both cell lines, but the subsequent step leading to the enzymatically highly active p17 product was blocked in the Bcl-2 overexpressing cells (Fig 2B). Caspase-9 cleavage was detected after 2–3 h of TRAIL treatment paralleling cytochrome c and Smac release in response to MOMP (Fig 1E) and this processing was significantly blocked after Bcl-2 overexpression. Notably, the onset of caspase-9 cleavage after Db-scTRAIL apparently precedes measurable bioactivity. We propose that caspase-9 cleavage occurs irrespective from its bioactivity that requires apoptosome formation [24,25].

### Caspase-9 plays a minor role only in TRAIL-induced apoptosis in NCI-H460 cells

**Fig 3:**
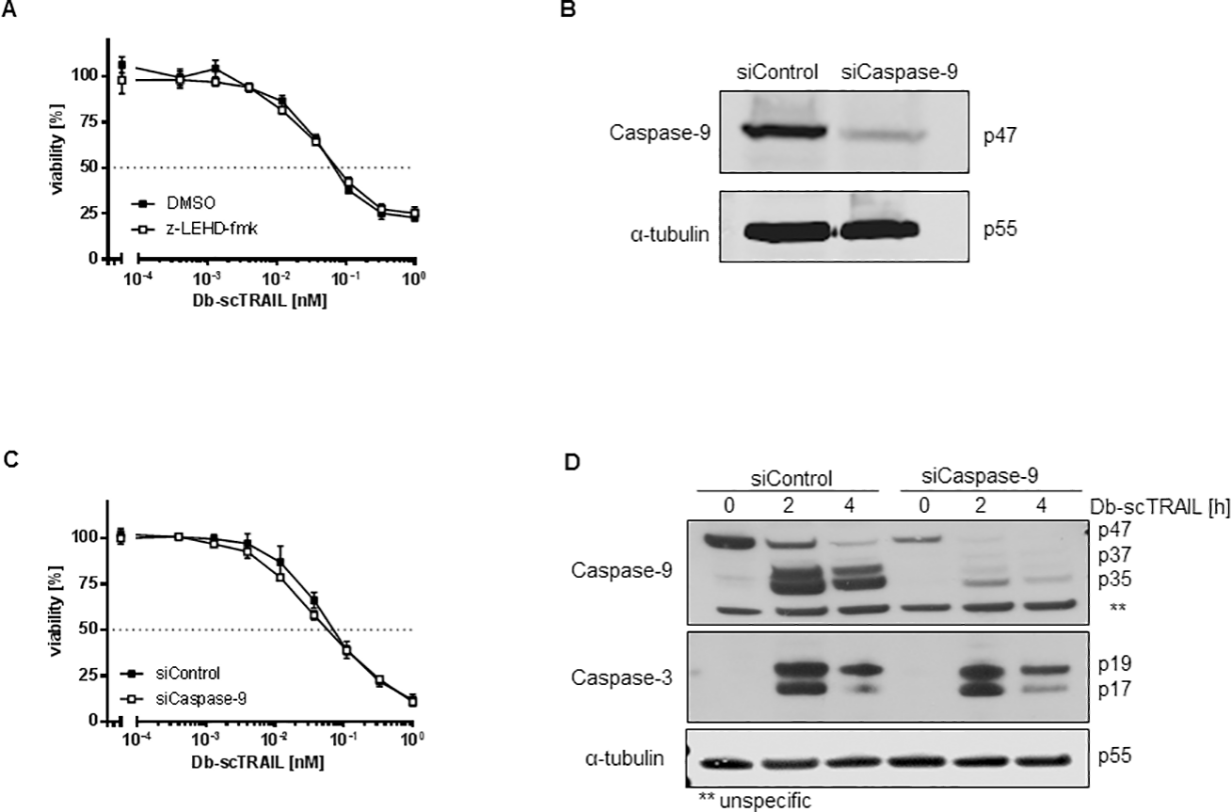
Caspase-9 plays only a minor role in TRAIL-induced apoptosis in NCI-H460 cells. (A) Cells were preincubated with caspase-9 inhibitor (z-LEHD-fmk, 50 μM) for 1 h, followed by stimulation with serial dilutions of Db-scTRAIL for 24 h. Viable cells were stained with crystal violet and absorbance was measured at 550 nm. (B) Western blot showing efficient downregulation of procaspase-9 by siRNA treatment. NCI-H460 cells were transfected with siRNAs (siControl and siCaspase-9) and harvested after 48 h. (C) NCI-H460 cells with downregulated caspase-9 do not show increased TRAIL resistance. Cells transfected with either non-targeting (siControl) or caspase-9 (siCaspase-9) siRNAs were stimulated 48 h post transfection with serial dilutions of Db-scTRAIL for further 24 h. (D) Cells pretreated as in (B) were treated for 2 and 4 h with Db-scTRAIL (1 nM), control cells were left untreated. Whole cell lysates were subjected to western blot analysis using antibodies specific for caspase-9 and caspase-3, α-tubulin was used to confirm equal loading.

In type II cells caspase-9 is believed to play an important role amplifying the caspase cascade in a mitochondria-dependent manner [26,27], although some publications have challenged this as a general rule [23,28–30]. Clearly, caspase-9 activity (Fig 2A) and cleavage (Fig 2B) were inhibited in the Bcl-2 overexpressing cells, where the mitochondrial pathway appears to be effectively inhibited as no cytochrome c release can be observed (Fig 1E). However, whether strong activation of caspase-9 observed in wildtype cells is causal for effective apoptosis or rather represents a bystander effect, was unclear. We therefore used the caspase-9-specific inhibitor z-LEHD-fmk and in addition downregulated caspase-9 using a siRNA approach. As presented in Fig 3A, z-LEHD-fmk conferred no significant protection of NCI-H460 cells over a broad concentration range of TRAIL. Similarly, although an effective downregulation of procaspase-9 could be achieved as demonstrated by Western blotting (Fig 3B), no significant effects on TRAIL sensitivity of these cells was noted (Fig 3C). In fact, comparable pattern of caspase-3 cleavage were observed in control siRNA-treated cells and cells with downregulated caspase-9 (Fig 3D). Although the caspase-9 cleavage pattern were hard to compare in the two cell lines due to the differential expression levels of the proenzyme, these data also suggest no major differences (Fig 3D). Together, these results strongly suggest that either very low amounts of caspase-9 are sufficient to effectively enhance apoptosis after mitochondrial depolarization or that caspase-9 plays no major role in this regard in NCI-H460 cells.

### Smac is an effective regulator of TRAIL-induced apoptosis in NCI-H460 cells

**Fig 4:**
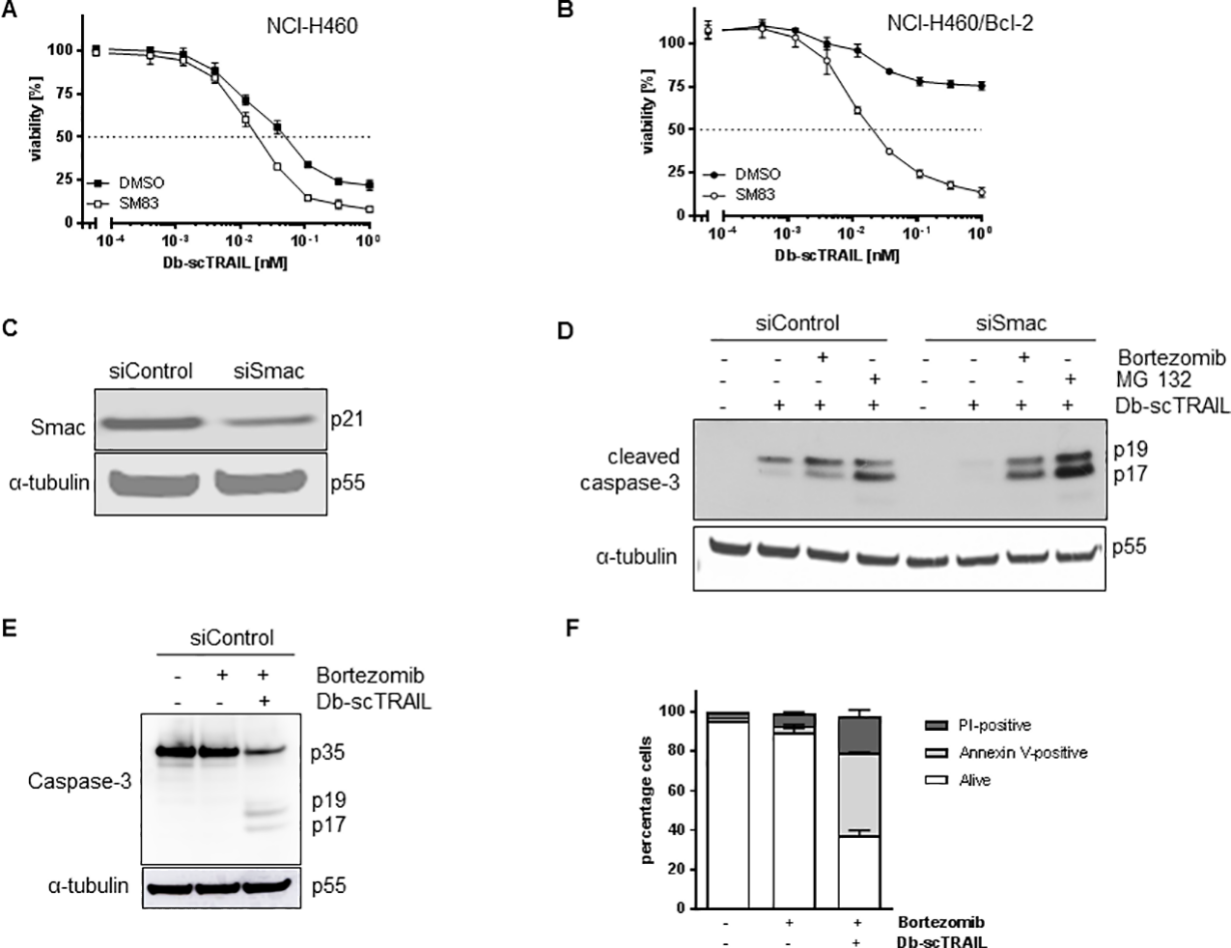
Smac is a potent regulator of TRAIL-induced apoptosis in NCI-H460 cells. (A) and (B) Smac mimetic SM83 sensitizes NCI-H460/Bcl-2 cells for TRAIL-induced apoptosis. NCI-H460 wild type (A) and NCI-H460/Bcl-2 cells (B) were preincubated with SM83 (1 μM) followed by stimulation with increasing concentrations of Db-scTRAIL for 24 h. Viable cells were stained with crystal violet and the absorbance was determined at 550 nm. Values from stimulated cells were normalized to unstimulated cells. Shown are mean values ± SD calculated from triplicates. One representative experiment out of three is shown. (C) Downregulation of Smac protein by siRNA treatment. NCI-H460 cells were transfected with siRNAs directed against Smac (siSmac) or non-targeting (siControl). Expression levels of Smac were determined 48 h later by western blotting. (D) Reduced caspase-3 processing after Smac downregulation is rescued by proteasome inhibition. Cells from (C) were preincubated with MG132 (25 μM) or Bortezomib (1 μM) followed by stimulation with Db-scTRAIL (1 nM) for 4 h. Immunoblotting was performed to detect cleaved caspase-3. (E) and (F) NCI-H460 cells were preincubated with Bortezomib (1 μM) followed by stimulation with Db-scTRAIL (1 nM) for 4 h. Immunoblotting was performed to detect cleaved caspase-3 (E) and apoptosis was analyzed by Annexin V-FITC/PI staining and flow cytometry (F).

A well characterized regulator of apoptosis released into the cytosol upon mitochondrial depolarization is the molecule Smac/DIABLO, representing an efficient antagonist for XIAP, but also inhibiting cIAP1 and cIAP2, thereby repressing their antiapoptotic activities [31]. Small molecule-based Smac mimetics have been designed based on the structure of the N-terminal tetrapeptide of native Smac, which are capable to bind a surface groove on the baculovirus repeat 3 (BIR3) domain of XIAP (reviewed in [32]). We used the Smac mimetic SM83, but TRAIL sensitivity of NCI-H460 cells was hardly affected by this molecule (Fig 4A). However, Bcl-2 overexpressing cells, showing strong protection against TRAIL-induced apoptosis, were efficiently resensitized to TRAIL by treatment with SM83 (Fig 4B). These data underscore the importance of mitochondrial depolarization in TRAIL-mediated cell death in NCI-H460 cells, pointing to the interplay between XIAP and Smac as a central regulatory circuit controlling final activation of caspase-3. We therefore downregulated Smac using a siRNA approach and investigated the level of caspase-3 activation. Not quite unexpected, an efficient downregulation of the Smac protein (Fig 4C) resulted in strongly reduced caspase-3 p19 and p17 levels in comparison to the control (Fig 4D). Cleavage products of caspase-3 are known substrates for XIAP ubiquitin ligase activity [33], consequently, we observed enhanced caspase-3 processing when we added the proteasome inhibitors Bortezomib or MG 132 (Fig 4D) while Bortezomib alone was insufficient for caspase-3-mediated cell death (Fig 4E and 4F).

### Overexpression of XIAP or of XIAP subdomains hardly affects TRAIL sensitivity

**Fig 5:**
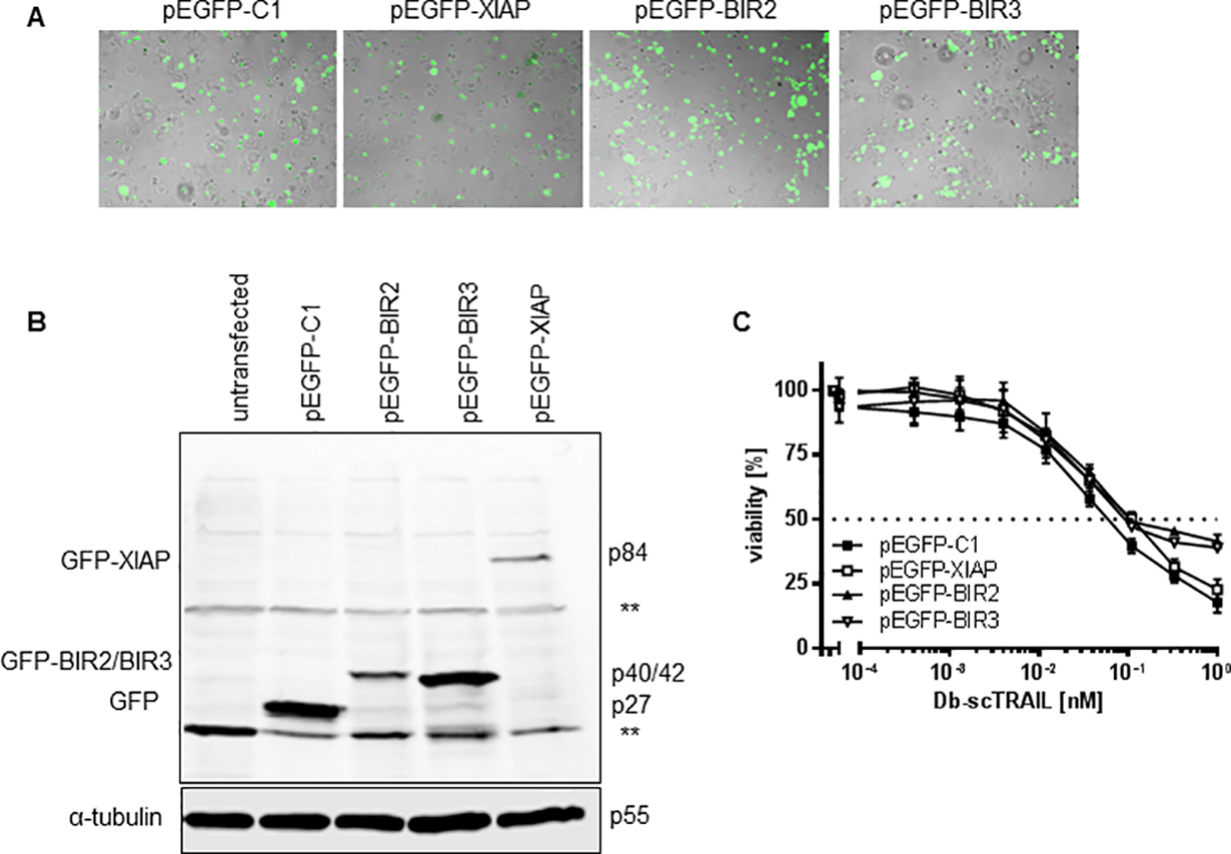
Overexpression of XIAP or XIAP subdomains hardly affect TRAIL sensitivity. (A) and (B) Transient transfection of XIAP and XIAP-derived BIR domains. NCI-H460 cells were transiently transfected with empty vector (pEGFP-C1) as a control or vectors encoding full length XIAP or the respective BIR2 and BIR3 domain. Transfection efficiency was checked by visualizing the GFP signal microscopically after 24 h (A). Whole cell lysates were run on a SDS-PAGE and immunoblot analysis was performed (B) using anti-GFP antibody. (C) Cells from (A) were stimulated with serial dilutions of Db-scTRAIL for 24 h. Viable cells were stained with crystal violet and the absorbance was determined at 550 nm. Cells transfected with pEGFP-C1 were used as control. Shown are mean values ± SD calculated from triplicates. One representative experiment out of three is shown.

Since the data obtained with the Smac mimetic SM83 and with downregulation of endogenous Smac protein pointed to a central role of the apoptosis inhibitor XIAP, we next overexpressed this molecule as well as the BIR2 and BIR3 subdomains thereof. Transfection efficiencies were somewhat variable, as controlled optically by immunoblotting, demonstrating the proper molecular weights of the respective GFP fusion proteins (Fig 5A and 5B). Compared to the relevant transfection control, no significant changes in TRAIL sensitivity could be observed, neither at lower, nor at high ligand concentrations used (Fig 5C). These results suggest that neither enhancement of the XIAP protein level nor expression of its subdomains BIR2 or BIR3 causes additional protection against TRAIL-mediated apoptosis. Alternatively, but according to our suggestions more unlikely, the transfection efficiencies were too low for significant effects visible in the whole cell population.

### XIAP downregulation restores TRAIL sensitivity in Bcl-2 overexpressing cells

**Fig 6:**
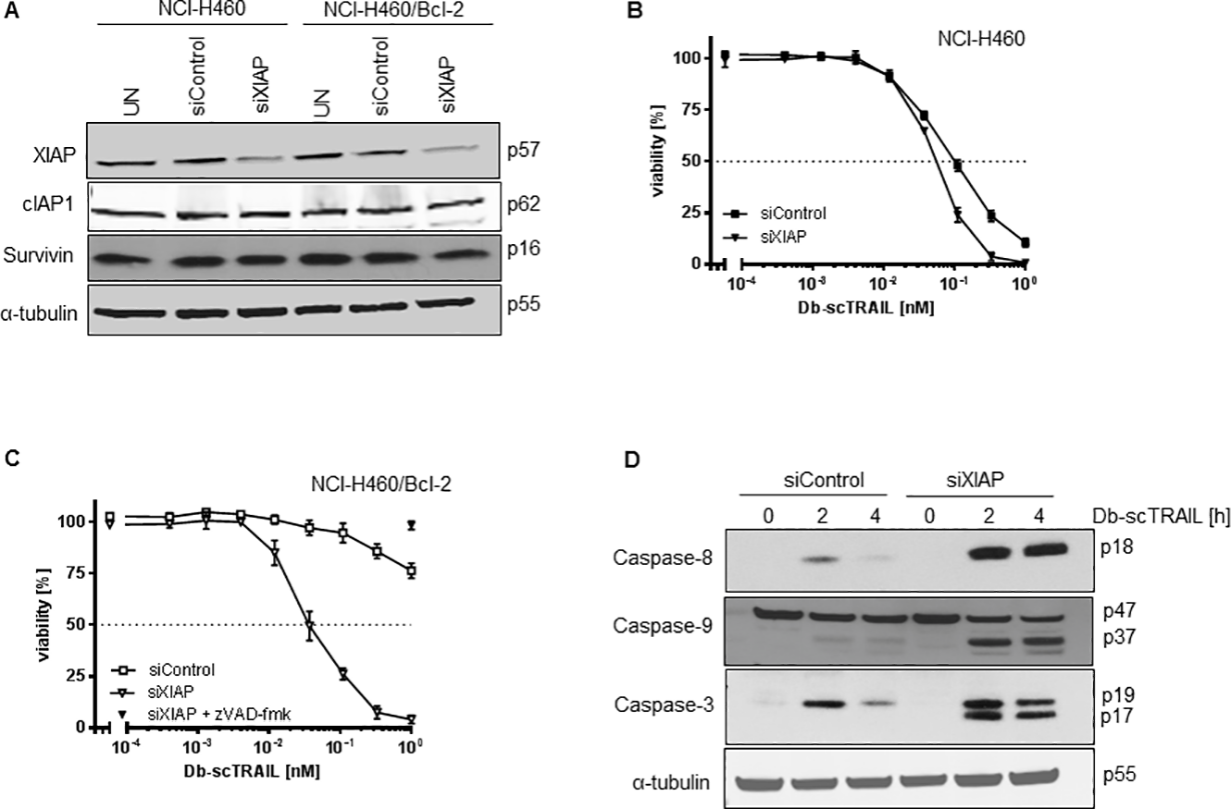
XIAP downregulation restores TRAIL sensitivity in Bcl-2 overexpressing cells. (A) Downregulation of XIAP by siRNA treatment. Cells were left untreated (UT) or were transiently transfected with non-targeting (siControl) or siRNA specific for XIAP (siXIAP). Protein expression was determined 48 h post transfection from whole cell lysates *via* immunoblot assay using an XIAP specific antibody. As control c-IAP1 and survivin were included. (B) and (C) NCI-H460 wild type (B) and NCI-H460/Bcl-2 cells (C) had been pretreated as in (A) were treated with serial dilutions of Db-scTRAIL (open symbols). NCI-H460/Bcl-2 cells transfected with siXIAP were incubated with 50 μM z-VAD-fmk and 1 nM Db-scTRAIL (filled triangle). After 24 h viable cells were stained with crystal violet. All values were normalized to those from unstimulated cells. The experiment was performed in triplicates and the data shown are representative of three independent experiments. (D) NCI-H460/Bcl-2 cells had been pretreated as in (A) were treated with Db-scTRAIL (1 nM) for 2 and 4 h and analyzed by immunoblotting using antibodies for cleaved caspase-8, -9 and -3. Tubulin-α was used as loading control. Blot shown is representative of three independent experiments.

As enhancement of the protein level of XIAP was not found to exert any significant effects on TRAIL sensitivity, we downregulated its endogenous level using a siRNA approach. Downregulation was efficient as shown in Fig 6A, whereas the protein levels of the two IAP family members cIAP-1 and survivin were unaffected. Downregulation of XIAP in fact resulted in some limited enhancement in TRAIL sensitivity in control NCI-H460 cells, visible in particular at higher ligand concentrations (Fig 6B). Notably, in the Bcl-2 overexpressing cells a very strong sensitization could be observed for these otherwise resistant cells (Fig 6C). The type of cell death was apoptosis, as the broad caspase inhibitor zVAD-fmk fully blocked TRAIL-induced apoptosis in NCI-H460/Bcl-2 cells with downregulated XIAP (Fig 6C). As expected, reduced XIAP levels were associated with enhancement of caspase processing (Fig 6D). For caspase-3, we observed the formation of fully processed p17 fragment from intermediate product p19 while this conversion was completely blocked in control-transfected cells. This indicated that cells expressing low XIAP levels may switch to a mitochondria-independent, i.e. type I cell response. Active caspase-3 p17 the in turn feeds back on caspase-8, resulting in formation of its fully processed p18 form and also on caspase-9 cleavage (Fig 6D).

### Mathematical modeling of the XIAP/Smac interplay in NCI-H460 cells

**Fig 7:**
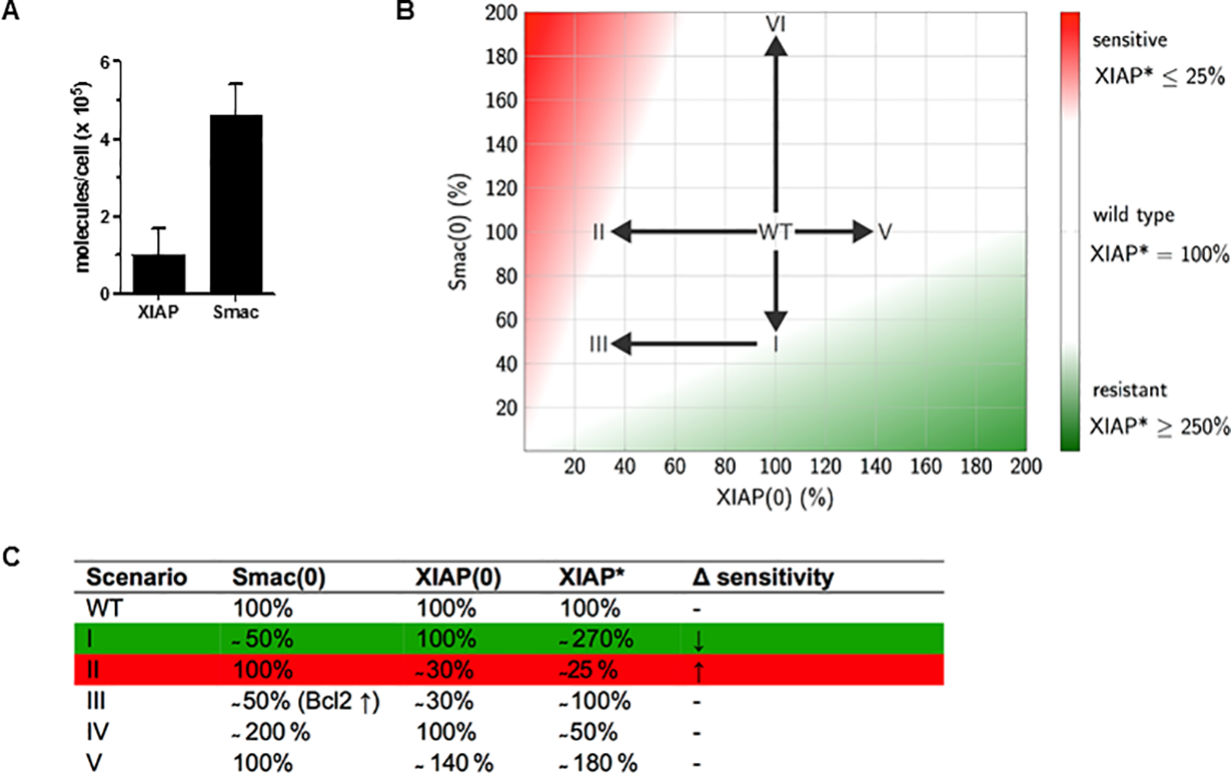
Mathematical modeling of the XIAP/Smac interaction in NCI-H460 in response to Db-scTRAIL. (A) The average numbers of XIAP and Smac in NCI-H460 in an unstimulated cell. XIAP and Smac molecules were quantified in NCI-H460 cells by western blotting. Mean ± SD is shown and were calculated from three independent experiments. (B) Regions of different cellular responses. Dependent on the initial amounts of XIAP and Smac, regions of enhanced or reduced apoptosis are sketched. Red regions represent XIAP* amounts lower or equal to 25% XIAP* compared to the wild type. Green color illustrates XIAP* amounts exceeding 250% of wild type. Different experimental scenarios (I-VI) are indicated by arrows. (C) Initial conditions of XIAP and Smac and the resulting steady state of XIAP are shown for the different scenarios. Green color indicates increased resistance to Db-scTRAIL, red color represents sensitivity, respectively.

The interaction of XIAP and Smac is believed to be pivotal for regulation of sensitivity to Db-scTRAIL. To analyze the consequences of variations in the amounts of these two proteins for the apoptotic response, we analyzed the XIAP/Smac complex formation mathematically, i.e. neutralization of XIAP and caspase-3 p19 processing. To this we determined the initial molecule numbers of XIAP and Smac in unstimulated NCI-H460 cells (Fig 7A) and analyzed the complex formation mathematically, based on reaction rates from literature [16]. As model output we chose the amount of Smac-free XIAP in steady state (XIAP*). Dependent on the respective amounts of XIAP and Smac, three different regions of the cellular status were defined: wild type (i.e. living cells which are TRAIL sensitive, colorless), ongoing cell death development (red) and TRAIL resistant property (green) (Fig 7B). Experimental conditions were allocated into this classification scheme. We assumed that cells become resistant to Db-scTRAIL treatment when XIAP* concentration exceeds that of wild type by a factor of 2.5. As a result, reducing Smac protein level by 50% would result in only a moderate resistance to TRAIL (scenario I, Fig 7B and 7C). This is in line with data from Smac siRNA experiment (Fig 4A) and Bcl-2 overexpression (Fig 4B) which acts mainly through a reduction of cytoplasmic Smac.

On the other hand, Db-scTRAIL sensitivity is assumed to increase when XIAP* falls below 25%. Accordingly, a reduction of XIAP to about 50% would lead only to slightly enhanced apoptosis (scenario II, Fig 7B), a prediction matching nicely our experimental data (Fig 6B). The combination of both reduced XIAP and Smac (scenario III) results in a wild type behavior which is in accordance with the experimental results shown in Fig 6C. The approach explains that moderate enhancement of either XIAP or Smac is not to affect the cellular phenotype (scenario IV and V), whereas very high amounts of Smac should lead to a sensitization. Taken together, our experimental data can be reconciled with the calculated XIAP* states.

## Discussion

The molecule TRAIL is capable to induce apoptosis in the human system by binding to two membrane receptors, TRAILR1 and TRAILR2. In NCI-H460 cells these two molecules are both expressed at the cell surface at near to equal levels, whereas the two antagonistic receptors TRAILR3 and TRAILR4 can be hardly detected. Effective interference of the antagonistic receptors with the respective agonists is therefore highly unlikely in these cells. Many tumor cells are sensitive to TRAIL treatment whereas normal cells typically display TRAIL resistance [10,34]. However, these differential cellular response patterns are by no means absolute, as e.g. artificially crosslinked TRAIL is strongly toxic for normal hepatocytes [35], and many tumor cells show strong TRAIL resistance [10]. In the present publication we used a targeted and multimeric form of TRAIL, engineered to possess high bioactivity, but still well tolerated *in vivo* [12]. A detailed analysis of the Db-scTRAIL-induced apoptotic signaling pathway in the NSCLC line NCI-H460 revealed the following four major findings.

First, Bcl-2 is capable to effectively protect NCI-H460 cells from TRAIL-induced apoptosis after only a moderate upregulation. This points to an important role of Bcl-2 in our model cell line and in fact abnormal expression of Bcl-2 has been reported in lung cancer than in adjacent non-cancerous tissues and Bcl-2 is discussed as a prognostic factor [36–39]. Further, Bcl-2 overexpression has been described to induce chemo- and radiotherapy resistance in NCI-H460 cells [40]. The Bcl-2 inhibitor Venetoclax (ABT-199) was recently approved for the treatment of chronic lymphocytic leukemia and is currently tested in several ongoing clinical trials to treat multiple hematological malignancies [41]. Bcl-2 represents a promising target also in lung carcinoma [42,43]. The combination of Venetoclax with inhibition of XIAP activity by Smac mimetics offers an attractive way to enhance death receptor-mediated apoptosis [44,45].

When we investigated the mitochondrial status in NCI-H460 cells we found a significant heterogeneity of the mitochondrial potential within the cellular population which could not be observed in six control cell lines derived from different tumors. Differences in mitochondrial activity have been linked to drug resistance [19,20]. However, we detected no direct effect of low or high mitochondrial potential on TRAIL sensitivity. Notably, Bcl-2 overexpressing NCI-H460 cells also showed a homogeneous and high mitochondrial membrane potential.

Second, Smac appears to be a central regulatory molecule in the mitochondrial amplification of caspase activation, rather than caspase-9. In accordance with these data apoptosis induced by chemotherapeutics in NCI-H460 cells was found to be mediated by caspase-8 and effector caspases and was dependent on activation of the mitochondrial pathway, but not on caspase-9 activation [13]. In fact, at least for some cell lines caspase-9 activation appears not to be required for death receptor-mediated apoptosis in a type II cell, although activation of the mitochondrial pathway was mandatory [28,29,46]. In general, however, caspase-9 is considered to be essential for apoptotic signaling through the mitochondrial pathway [47]. However, the exact role of caspase-9 cleavage and activity during apoptosis remains questionable [24,48]. Clearly, Smac is a multifunctional molecule capable to interact with the BIR3 of XIAP for direct inhibition, but also favoring proteasomal degradation. Accordingly, Smac has been suggested to efficiently promote apoptosome-mediated activation of effector caspases [49]. On the other side Smac-deficient mice appear healthy and cell lines derived thereof showed normal apoptotic responses to some stimuli although caspase-3 activation induced by cytochrome c was clearly inhibited [50].

Third, the molecule XIAP obviously plays a pivotal role controlling the apoptotic machinery far at the end of the caspase cascade. This has been already suggested from the described importance of Smac, representing a direct antagonist of this molecule. It therefore appears likely that control of effector caspases by the two opposing molecules Smac and XIAP represents the final hurdle before the cell becomes dismantled by a strong activation of effector caspases. These results are in accordance with those from Jost and colleagues, who found XIAP to be a central regulator in CD95/Fas-induced apoptosis, controlling whether a cell responds as a type I or a type II cell [32]. Similarly, cooperativity between XIAP inhibitors and TRAIL resulted in a strong apoptotic response in pancreatic carcinoma or leukemia cells, capable to even overcome a Bcl-2-mediated resistance [51,52].

Focusing on the complex formation of XIAP and Smac, a mathematical description of the reversible binding reaction reproduces our experimental findings. As the molecule number of Smac is in excess compared to XIAP, moderate downregulation of Smac is predicted to hardly affect TRAIL sensitivity. In addition, the effect of downregulated Smac can be overcome by XIAP downregulation. Definition of regions of sensitivity and resistance in dependence of the initial amount of XIAP and Smac may help to classify and design further experimental setups.Fourth, interesting pattern in the sequential activation of caspase-3 were detected, all indicating that control of the caspase cascade by Bcl-2 is located late in this processes of sequential caspase cleavage. In particular initiator caspase-mediated initial cleavage of caspase-3 between the large and small subunits to generate the p19 fragment occurred with similar kinetics in TRAIL sensitive wildtype NCI-H460 cells and the Bcl-2 overexpressing resistant variant (Fig 2B). In contrast, the final autocleavage step to generate the fully active p17 fragment of caspase-3 was blocked in Bcl-2 overexpressing cells (Fig 2B). A likely explanation for this result argues that XIAP is capable to effectively inhibit caspase-3 maturation in NCI-H460/Bcl-2 cells, whereas the initial caspase-3 cleavage step can occur unhampered in the DISC. The fact that caspase-8 cleavage occurs significantly stronger in wild-type than in Bcl-2 overexpressing cells (Fig 2B) is therefore likely the consequence of a positive feedback loop leading from active effector caspases to caspase-8, thereby further enhancing activation of the caspase cascade [23]. The initial cleavage step of caspase-8 leading to formation of the p41/p43 product appears to occur at comparable levels in wild-type and Bcl-2 overexpressing cells (Fig 2B), which can be easily explained by its location in the DISC, unaffected by the consequences of Bcl-2 overexpression. Strong caspase-9 activation and cleavage can be observed in cells where the mitochondrial pathway becomes activated (Fig 3F), although our results question the importance of this activation step for the apoptotic event (see above).

Together, our results strongly suggest an interesting aspect of the mitochondrial apoptotic pathway. In type II cells its activation is mandatory for execution of the final apoptotic program. In cells, where caspase-9 activity is indispensable for execution of the apoptotic program, surviving cells might stay in a status with only weakly activated caspase cascade. In NCI-H460 cells in contrast, where Smac but not caspase-9 activity appears to be mandatory for induction of cell death, surviving cells are likely to have a strongly activated caspase cascade, controlled at the last step of caspase-3 activation. These results are of particular interest with a view to recent data showing that sublethal caspase activity allows cells to escape from apoptosis, but surviving cells accumulate DNA damage and rather promote tumor development [53,54].

## Materials and methods

### Maintenance of cell lines and cytotoxicity assay

The cell lines NCI-H460, HeLa and HCT-116 were obtained from ATCC (Manassas, USA). Cells were cultured in RPMI-1640 medium supplemented with L-glutamine and 5% fetal calf serum (FCS) and kept at 37°C with 5% CO_2_ and 96% relative humidity. Transfectants of NCI-H460 cells stably overexpressing Bcl-2 (NCI-H460/Bcl-2) were supplemented with culture medium containing 3 μg/ml puromycin. For cytotoxicity assays 15000 cells per well were plated in 96-well flat bottom plate. Next day cells were treated with serial dilutions of Db-scTRAIL starting with 1 nM. Cell viability was determined by crystal violet staining after 24 h and absorbance was measured in a microplate reader at λ = 550 nm (Infinite M200, Tecan). All values were normalized to those from unstimulated wells. Alternatively, cells were harvested, stained with Annexin V-FITC and propidium iodide (PI) and cell viability was determined by flow cytometry (MACSQuant®, MiltenyiBiotec, Germany).

### Antibodies and reagents

Following antibodies were used: Rabbit polyclonal anti-Bcl-2, rabbit monoclonal anti-caspase-3 (8G10), mouse monoclonal anti-caspase-8 (1C12), anti-Smac/DIABLO, rabbit polyclonal anti-caspase-9, rabbit monoclonal anti-c-IAP1 (D5G9) and rabbit monoclonal anti-Survivin (71G4B7) antibodies were purchased from Cell Signaling Technology (Danvers, MA, USA). Mouse monoclonal anti-XIAP and mouse monoclonal anti-cytochrome c were from BD Pharmingen (Heidelberg Germany). Goat polyclonal anti-β-actin was from Santa Cruz Biotechnology (California, USA), anti-α-tubulin was purchased from Sigma-Aldrich (Taufkirchen, Germany). Mouse monoclonal anti-GFP was from Roche Diagnostics (Mannheim, Germany). Mouse monoclonal antibodies anti-TRAIL receptors 1–4 were from R&D Systems (Minneapolis, Minnesota, USA), mouse anti-human EGFR from BioLegend (San Diego, USA). Corresponding mouse IgG1 and IgG2b isotype control antibodies were obtained from BioLegend and phycoerythrin-conjugated goat anti-mouse IgG+IgM was from Dianova (Hamburg, Germany). Caspase substrates, Ac-DMQD-AMC (caspase-3), Ac-IEPD-AMC (caspase-8) and Ac-LEHD-AMC (caspase-9) were from Enzo Life Sciences, Switzerland. Caspase-9 inhibitor z-LEHD-fmk was from R&D systems and Bcl-2 family inhibitor ABT-737 was from Santa Cruz Biotechnology (California, USA). TMRM was from Thermo Fisher Scientifiy (USA) and Annexin V-FITC/PI was obtained from BD Pharmingen. Diabody-single chain TRAIL directed against EGFR (DbαEGFR-scTRAIL), shortly termed Db-scTRAIL [12], was kindly provided by Dr. Martin Siegemund, Institute of Cell Biology and Immunology, University of Stuttgart, Germany. Stock solutions of Db-scTRAIL were freshly diluted with culture medium to the indicated final concentration for each experiment. Smac mimetic SM83 was produced as described before [55].

### Caspase activity assay

Briefly, cells from both cell lines (NCI-H460 and NCI-H460/Bcl-2) were stimulated with 1 nM Db-scTRAIL for the indicated times, washed with PBS, harvested and pelleted. Cells were then lysed using caspase lysis buffer with freshly added protease inhibitor cocktail (Roche Diagnostics, Germany) incubated on ice and centrifuged at 13000 × g at 4°C for 10 min. Protein concentration was determined by Bradford assay. 4 μg of total protein was used to determine caspase-3 activity, while 32 μg were used for caspase-8 and 20 μg for caspase-9 activity determination. Cell lysates were transferred to the wells of a black 96-well plate and mixed with caspase activity buffer (10 mM HEPES pH 7.0, 220 mM mannitol, 68 mM sucrose, 2 mM NaCl, 2.5 mM KH_2_PO_4_, 0.5 mM EGTA, 2 mM MgCl_2_ 5 mM pyruvate, 1mM DTT), containing the appropriate caspase substrate. The changes in fluorescence intensity were detected using a microplate reader each 2 min for 2 h at 37˚C. Enzymatic activities were calculated from the slopes of the resulting lines and normalized to the highest value.

### Cell lysis and subcellular fractionation

Cells were stimulated, harvested and pelleted by centrifugation, washed with PBS and resuspended in lysis buffer (20 mM Tris-HCl pH 7.5, 150 mM NaCl, 1 mM Na_2_EDTA, 1 mM EGTA, 1% (v/v) Triton X-100) with freshly added protease inhibitor. After 15 min incubation on ice, samples were centrifuged at 13000 × g at 4°C for 10 min. Protein concentration was determined by Bradford assay. For subcellular fractionation, cells stimulated for the indicated time points were collected, and lysed in a permeabilization buffer containing 200 μg/ml digitonin for 5 min. Plasma membrane permeabilization was checked by trypan blue staining and the lysate after centrifugation for 10 min at 2000 × g was used as mitochondrial fraction. The pellet was resuspended in lysis buffer containing 1% Triton-X100 and incubated on ice for 15 min, centrifuged at 13000 × g for 10 min and supernatants were transferred to new reaction tubes.

### Western blotting

Cell samples were separated by SDS-PAGE and transferred onto nitrocellulose membrane. Membranes were probed with horseradish peroxidase-conjugated α-mouse IgG or α-rabbit IgG secondary antibodies (Dianova, Hamburg, Germany) and immunodetection was performed using the ECL system (Thermo Fisher Scientific, USA). Membranes were stripped with 0.2% NaOH before reprobing using different antibodies.

### Flow cytometry

For intracellular staining of Bcl-2, cells were harvested, pelleted and fixed with 4% paraformaldehyde. After washing with PBA (PBS supplemented with 0.05% (w/v) BSA and 0.02% (w/v) NaN_3_), cells were permeabilized with FACS^TM^ permeabilization solution 2 (BD Pharmingen, Germany), and incubated with primary antibody diluted in PBA supplemented with 10% FCS for 60 min on ice followed by two washing steps. Cells were then incubated with PE-labeled secondary antibody diluted in PBA supplemented with 10% FCS and subsequently analyzed by flow cytometry (MACSQuant®, MiltenyiBiotec, Germany).

For staining of EGFR and TRAIL receptors, cells were resuspended in PBA containing primary antibody. After incubation for 45 min on ice, cells were washed in PBA and incubated with PE-conjugated secondary antibody in PBA. After 45 min of incubation cells were washed again and analyzed by flow cytometry. TMRM was used to analyze the mitochondrial membrane potential. Cells were loaded with 60 nM TMRM diluted in cell culture media for 30 min, harvested, washed with PBA and analyzed by flow cytometry.

### Time-lapse microscopy

NCI-H460 cells were cultivated on 35 mm glass bottom dish. Next day, medium was replaced by phenol red-free medium containing 5% FCS and 60 nM TMRM and the dish was placed in the incubation chamber (37°C, 5% CO_2_) of the Zeiss Cell Observer microscope (Zeiss, Germany) equipped with a 20x objective and a CCD camera. Apoptosis was induced with 1 nM Db-scTRAIL and cells were then imaged in 15 min interval for 6 h. TMRM was excited using the 587 nm laser and fluorescence was detected at 610 nm. Image analysis was done with Zen Blue software (Zeiss). TMRM intensity, corresponding cell size and the time from Db-scTRAIL addition to apoptotic cell death were analyzed for 100 cells. Cells were classified as apoptotic at the time of first appearance of membrane blebbing and cell shrinkage, typical signs of apoptosis.

### Cloning of BIR domains

The gene sequences encoding full length XIAP, the BIR2 (aa 126–229) or BIR3 (aa 244–352) domains were amplified using appropriate primers from plasmid pcDNA3.1-myc-XIAP (kindly provided by D. Kulms, Dresden, Germany) and cloned into pEGFP-C1 (Clontech) using *Eco*RI and *Xho*I sites. Resulting expression constructs were verified by DNA sequencing (GATC Biotech AG, Konstanz, Germany).

### Transfections

For transient expression of full length XIAP and its BIR domains, cells were nucleofected using Amaxa Nucleofector^TM^ Kit T (Lonza, Switzerland) according to the manufacturer’s instructions. Transfection efficiency was checked after 24 h by GFP positive cells under the microscope and afterwards cells were harvested and analyzed for the protein levels of XIAP and BIR domains by immunoblotting using anti-GFP antibody. Cells were transfected with either control or gene-specific siRNAs from Silencer^®^ Select (Fisher Scientific GmbH, Germany) at a final concentration of 8 nM using DharmaFECT (Dharmacon, GE healthcare, UK) according to manufacturer’s instructions. Protein expression levels were analyzed *via* western blotting 24 h post transfection.

### Quantification of initial concentration of XIAP and Smac in NCI-H460

The cellular molecule number of XIAP was determined by quantitative western blotting. First, HeLa cells were transfected with plasmid GFP-XIAP using Lipofectamine LTX & Plus (Life technologies). Cells were lysed 48 h later and increasing amounts of total cell lysates were subjected to western blotting in parallel with increasing amounts of recombinant purified GFP and different amounts of cell lysates of NCI-H460 cells. Anti-GFP and anti-XIAP were used for detection of GFP, GFP-XIAP or XIAP, respectively. GFP-XIAP intensities were compared with purified GFP, and the XIAP amount in HeLa and NCI-H460 cells was calculated from GFP-XIAP intensities, respectively. We consider the unit molecule numbers/cell more illustrative than the unit molar and used an average cell volume of 3.1 pL [56]. Three experiments were evaluated. Smac molecule number was determined by comparative quantitative western blotting. HCT-116 cells were used as reference cell line [57]. Total cell extracts from NCI-H460 and HCT-116 cells were compared for their XIAP intensity by western blotting. Three experiments were analyzed.

### Mathematical model and implementation

To validate the experimental results, a minimal mathematical model was analyzed. The model consists of three ordinary differential equations that describe the reversible binding of Smac and XIAP [16]. Therefore, we used the initial conditions of Smac and XIAP for NCI-H460. As model output, steady state values of Smac-free XIAP (XIAP*) were calculated in dependence on different initial amounts of Smac and XIAP, representing experimental conditions. XIAP* represents the relevant amount of XIAP blocking the processing of activated caspase-3 p19 form. All calculations were performed with MATLAB release 2017a (The MathWorks, Inc., Natick). First, the amount of XIAP* was calculated for the wildtype scenario. This value serves as reference value and was set to 100%. XIAP* values derived from scenario I and II were used for determining threshold values of XIAP*. Exceeding or deceeding the specific threshold implies increased resistance or sensitivity, respectively. The remaining scenarios were classified by their XIAP* levels and compared to experimental data.

## Supporting information

S1 Fig Expression of apoptosis-related proteins is not altered by Bcl-2 overexpression in NCI-H460 cells. (A) Flow cytometric analysis of TRAIL receptor cell surface expression in NCI-H460 or NCI-H460/Bcl-2 cells. TRAILR1-4 were immunostained with mouse anti-TRAILR1-4 antibodies (black histogram) or the respective isotype control antibodies (grey histogram) followed by incubation with anti-mouse IgG-PE conjugated secondary antibody. (B) Cell surface expression of EGFR (red histogram) in NCI-H460 or Bcl-2 overexpressing cells, isotype control is shown as black histogram. (C) Total cell extracts of NCI-H460 or NCI-H460/Bcl-2 were immunoblotted using antibodies directed against Bcl-2, caspase-8, caspase-3 or XIAP. Tubulin-α was used as loading control. Blots shown are representative of three independent experiments. (D) NCI-H460 or Bcl-2 overexpressing cells were stained with 60 nM TMRM and analyzed by flow cytometry. (E) NCI-H460 cells loaded with TMRM were treated with Db-scTRAIL (1 nM) and imaged by live-cell fluorescence microscopy. Apoptotic cell death time values and respective cellular TMRM intensities were analyzed for randomly chosen cells (n = 100).
(TIF)
