# Supplementary figures and images for: Bcl-2-mediated control of TRAIL-induced apoptotic response in the non-small lung cancer cell line NCI-H460 is effective at late caspase processing steps
Source: PLoS One. 2018 Jun 21;13(6):e0198203. doi: 10.1371/journal.pone.0198203 (PMC6013189; doi:10.1371/journal.pone.0198203)

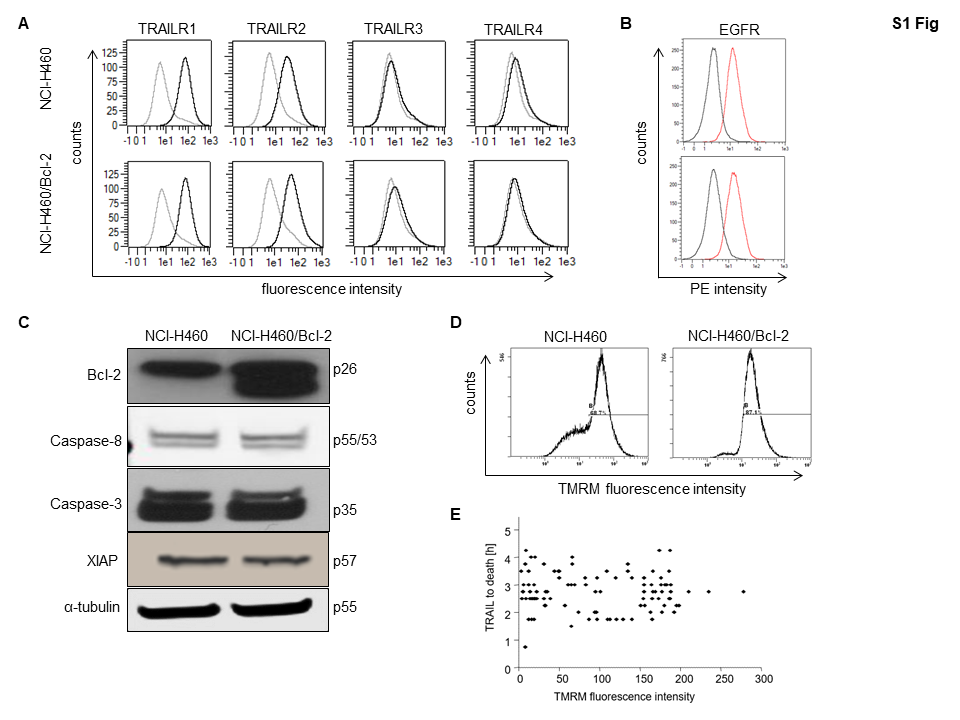

Supplement: S1 Fig — (A) Flow cytometric analysis of TRAIL receptor cell surface expression in NCI-H460 or NCI-H460/Bcl-2 cells. TRAILR1-4 were immunostained with mouse anti-TRAILR1-4 antibodies (black histogram) or the respective isotype control antibodies (grey histogram) followed by incubation with anti-mouse IgG-PE conjugated secondary antibody. (B) Cell surface expression of EGFR (red histogram) in NCI-H460 or Bcl-2 overexpressing cells, isotype control is shown as black histogram. (C) Total cell extracts of NCI-H460 or NCI-H460/Bcl-2 were immunoblotted using antibodies directed against Bcl-2, caspase-8, caspase-3 or XIAP. Tubulin-α was used as loading control. Blots shown are representative of three independent experiments. (D) NCI-H460 or Bcl-2 overexpressing cells were stained with 60 nM TMRM and analyzed by flow cytometry. (E) NCI-H460 cells loaded with TMRM were treated with Db-scTRAIL (1 nM) and imaged by live-cell fluorescence microscopy. Apoptotic cell death time values and respective cellular TMRM intensities were analyzed for randomly chosen cells (n = 100). (TIF) [file pone.0198203.s001.tif]
